# Dermatomyofibromas harbor PDGFRB mutations – another tyrosine kinase-driven neoplasm

**DOI:** 10.1007/s00428-025-04128-z

**Published:** 2025-05-19

**Authors:** Uta Flucke, Laura S. Hiemcke-Jiwa, Joost M. van Gorp, Don Hayes, Marieke M. B. Seyger, Marco J. Koudijs, Lennart A. Kester, Sjoerd van Helvert, Remco T. P. van Cruchten

**Affiliations:** 1 Department of Pathology, Radboud University Medical Center, P.O. Box 9101, 6500 HB Nijmegen, The Netherlands; 2 Princess Máxima Center for Pediatric Oncology, Utrecht, The Netherlands; 3 Department of Pathology, University Medical Center Utrecht, Utrecht, The Netherlands; 4 Department of Pathology, St Antonius Hospital, Nieuwegein, The Netherlands; 5 Department of Pathology, Meander Medical Center, Amersfoort, The Netherlands; 6 Department of Dermatology, Radboud University Medical Center, Nijmegen, The Netherlands

**Keywords:** Dermatomyofibroma, Mesenchymal tumors, Skin, PDGFRB, Protein kinase, Mutational analysis

## Abstract

Platelet-derived growth factor receptor beta (PDGFRB) is one of the numerous members of the receptor tyrosine kinase protein family. When altered, it is known to be the driver mutation in different mesenchymal neoplasms, such as pericytic tumors, inflammatory myofibroblastic tumor, and sarcomas with myogenic differentiation. We investigated seven dermatomyofibromas for the presence of a *PDGFRB* mutation. Patients were 6 females and 1 male. Ages ranged from 2 to 59 years. Neoplasms were located in the shoulder (2), neck (2), upper arm (1), knee (1), and calf (1). Clinically, they appeared as ill-defined plaques. Complete excision was performed in four cases. In three cases, only a biopsy was taken. Histomorphologically, these dermal ill-defined tumors consisted of fascicles of slender myofibroblastic cells oriented often parallel to the epidermis. Their nuclei were monomorphic and elongated, and the cytoplasm was inconspicuous. Involvement of the superficial subcutis was seen in four cases. Immunohistochemically, neoplasms expressed SMA (5/7), focally desmin (1/5), and CD34 (4/6), while S100 was lacking (0/7). By DNA or RNA sequencing, *PDGFRB* activating mutations were identified in 6/7 tumors. Four neoplasms harbored a mutation in exon 12 encoding for the juxtamembrane domain and 2 neoplasms in exon 14 encoding for the tyrosine kinase domain. Sequencing analyses results highlight that these benign skin tumors belong to the broad spectrum of tyrosine kinase-driven neoplasms.

## Introduction

Protein kinase–related mesenchymal tumors, mostly driven by activating alterations in receptor tyrosine kinases, are an emerging and rapidly growing group comprising benign, locally aggressive, and malignant neoplasms [1–4].

Tumors are situated superficially (skin and subcutis), in deep soft tissue and bone, or viscerally, with the anatomical site being one important management parameter. Affected patients show a broad age range, but it seems that younger patients are more often involved [1, 3, 5–9].

The morphological, immunohistochemical, and genetic spectrum is broad; however, there is considerable overlap, resulting in several diagnostic hallmarks: The cell type is often (myo)fibroblastic with monomorphic nuclei. Cells are embedded in a fibromyxoid/hyaline matrix, often possessing prominent intermediate-sized (staghorn-like) vessels [1–4, 10].

The immunohistochemical profile is dominated by the expression of CD34 and S100 but is inconsistent with a very heterogeneous marker profile that can be confusing [2–4, 8–10].

From the genetic point of view, many hyperactivated protein kinases are known to be the driver mutations in such neoplasms [1, 3, 4, 8–10].

Platelet-derived growth factor receptor beta (PDGFRB) and its paralog alpha are two of the numerous members of the receptor tyrosine kinase (RTK) protein family. They belong to the class III family along with c-KIT, colony-stimulating factor 1 receptor (CSF1R), and Fms-like tyrosine kinase 3 receptor (FLT3). When altered, *PDGFRA* and *PDGFRB* play a role in various disorders, including soft tissue tumors [11, 12]. Gene fusions involving *PDGFRB* are reported in inflammatory myofibroblastic tumors, with *PDGFRA* being alternatively altered, in lipofibromatosis and in myofibromatosis [13–16]. *PDGFRB* gain-of-function mutations have been identified in pericytic tumors harboring alternatively or rarely simultaneously *NOTCH3* mutations with a similar biological effect [11, 17–19].

Based on an index case, we found recurrent *PDGFRB* gain-of-function mutations as the oncogenic driver in a cohort of dermatomyofibromas, adding these neoplasms to the protein kinase-related tumors.

## Material and methods

Tumors were retrieved from our files. All samples were fixed in 4% buffered formalin, routinely processed, and embedded in paraffin (FFPE). Four-µm-thick sections were stained with hematoxylin and eosin. Patient information was provided by the physicians.

Samples were handled according to the ethical guidelines described in “Code for Proper Secondary Use of Human Tissue in the Netherlands” in a coded (anonymized) manner, as approved by the local institutions.

### Immunohistochemistry

Four-µm-thick sections were stained using an automated Dako Omnis stainer (Agilent/Dako). Pretreatment was performed with retrieval solution high pH from DAKO. The following antibodies were used: CD34 (clone, Qbend 10), SMA (clone, 1 A4), desmin (clone, D33), and S100 (polyclonal rabbit). All antibodies were ready to use, provided by Dako.

### DNA mutation analysis

After macrodissection of regions with the highest content of neoplastic cells from FFPE tissue sections (generally 10 sections of 6 µm), genomic DNA was isolated as described in Eijkelenboom et al. [20], using 5% Chelex-100 and 20 mg/ml proteinase K, and DNA concentrations were measured using the Qubit 3.0 Fluorometer with the broad range kit (Q32853; Thermo Fisher). For cases 3, 4, and 7, targeted amplification of *PDGFRA* (NM_006206.6) exon 12, 14, and 18 (codons 552–595, 639–727, and 822–862) and *PDGFRB* (NM_002609.4) exons 11, 12, 14, 15, 18, and 23 (codons 528–602, 639–727, and 822–862) was performed using single-molecule molecular inversion probes (smMIPs) as described in Eijkelenboom et al. [20]. For case 1, the complete coding sequence of *PDGFRA* and *PDGFRB* was analyzed via enrichment using the Illumina Trusight Oncology 500 panel as described in Kroeze et al. [21]. For cases 2 and 6, the complete coding sequence of *PDGFRA* and *PDGFRB* was analyzed using whole exome sequencing based on the Twist Comprehensive Exome 2.0. Alignment of exome data was performed using bwa-mem2 and variant calling using Mutect2 [22, 23]. All raw data is available upon request. For all cases, next-generation sequencing was performed on an Illumina NextSeq500 or Novaseq6000 system.

### Whole transcriptome/mRNA sequencing

In case 5, RNA was isolated from FFPE material by standard protocol using the AllPrep DNA/RNA/Protein FFPE Kit (Qiagen). Libraries were generated with 300 ng RNA using the KAPA RNA HyperPrep Kit with RiboErase (Roche) and subsequently sequenced on a NovaSeq 6000 system (2 × 150 bp) (Illumina). RNA sequencing data were processed as per GATK 4.0 best practices workflow for variant calling using a wdl and Cromwell-based workflow (https://gatk.broadinstitute.org/hc/en-us/sections/360007226651-Best-Practices-Workflows). This included performing quality control with Fastqc (version 0.11.5) to calculate the number of sequencing reads and the insert size, Picard (version 2.20.1) for RNA metrics output, and MarkDuplicates [24]. The raw sequencing reads were aligned using Star (version 2.7.0f) to GRCh38 and gencode version 29 [25].

## Results

Clinical, immunohistochemical, and molecular results are presented in Table 1.

**Table 1:** Clinical, immunohistochemical, and molecular data

Case	Sex/Age	Site	Size (cm)	Clinical procedure and follow-up	IHC	*PDGFRB*-Mutation
1	f/10y	Neck		Biopsy No progression (18 months)	SMA -, CD34 ±, S100 -	Exon 12: c.1757_1777 del p.(Q586_W593 delinsR) VAF: 18% Juxtamembrane domain
2	f/24y	Upper arm, dermis	1.2	Excision R0	SMA -, desmin, -/+, CD34 ±, S100 -	Exon 12: c.1763_1777 del p.(P588_W593 delinsR) VAF: 27% Juxtamembrane domain
3	f/23y	Shoulder	0.7	Excision R0	SMA ±, desmin -, CD34 -, S100 -	Exon 14: c.1996 A > T p.(N666Y) VAF: 15% Tyrosine kinase domain
4	m/2y	Neck		Biopsy No follow-up	SMA ±, desmin -, CD34 -, S100 -	Exon 14: c.1998 C > A p.(N666 K) VAF: 32% Tyrosine kinase domain
5	f/22y	Knee (back)	2	Excision R0	SMA -/+, desmin -, CD34 -/+, S100 -	Exon 12: c.1706_1736 delinsGACT p.(I569_Y579 delinsRL) VAF: 15% Juxtamembrane domain (RNA seq)
6	f/20y	Shoulder	2.3	Excision R0	SMA ±, desmin -, CD34 ±, S100 -	Exon 12: c.1685 A > G p.(Y562 C) VAF:15% Juxtamembrane domain
7	f/59	calf		Biopsy No progression (13 y)	SMA +, S100 -	None

Abbreviations: *f*, female; *m*, male; *y*, year; *VAF*, variant allele frequency

Seven tumors from six females and one male were collected. The age of the patients ranged from 2 to 59 years (median, 22 years; mean, 23 years). Neoplasms originated in the shoulder (*n* = 2), neck (*n* = 2), upper arm (*n* = 1), back of the knee (*n* = 1), and calf (*n* = 1). Clinically, tumors appeared as ill-defined, slightly erythematous, and hyperpigmented plaques. Excision was performed in four cases, and a biopsy only was taken in three cases. There were no recurrences of the excised neoplasms. The tumors with biopsy only were stable (*n* = 2); one patient was lost to follow-up.

**Fig. 1:**
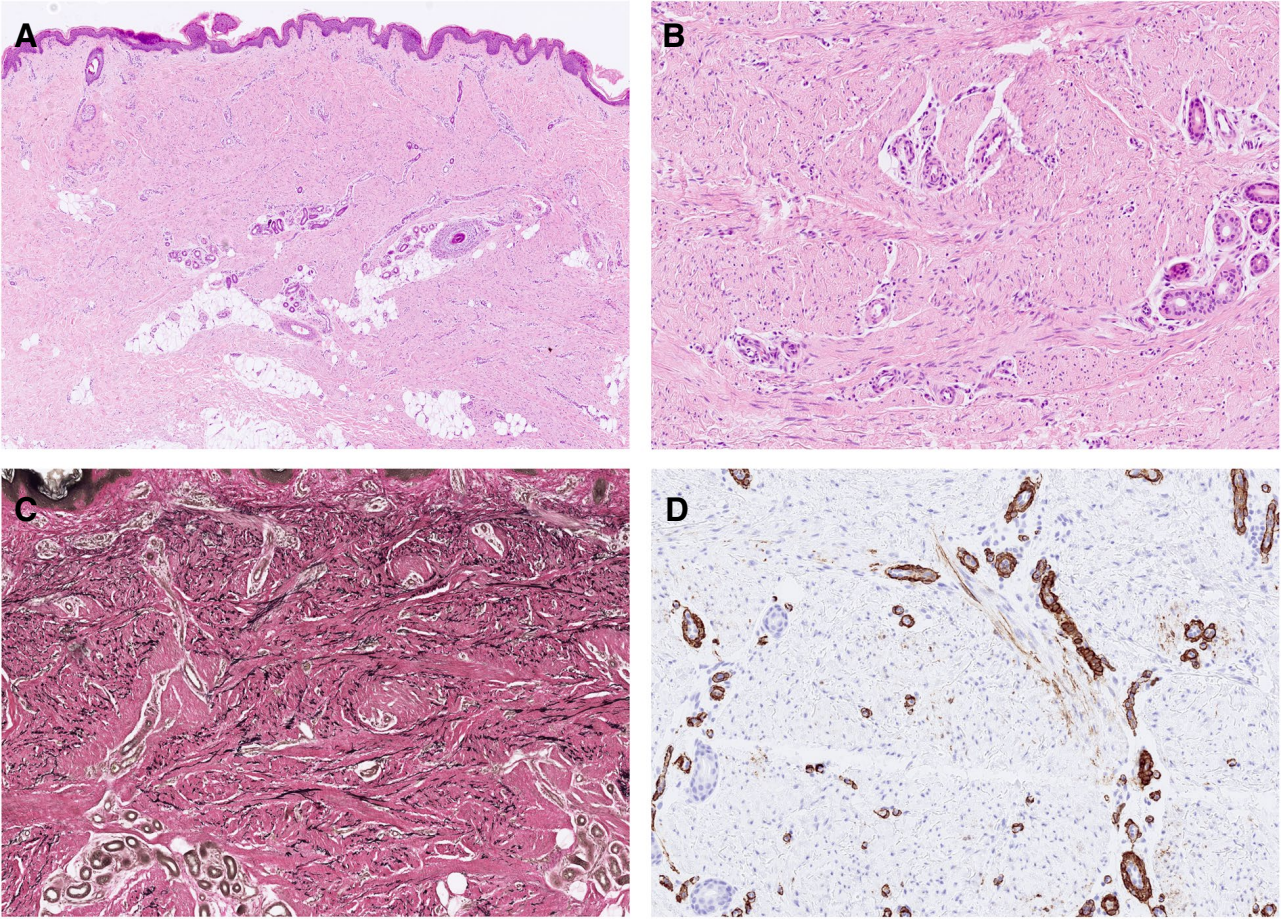
Some of the tumors infiltrated superficially the subcutis (**A**). They consisted of monomorphic, slender myofibroblastic cells arranged in bundles mainly parallel to the skin surface (**A**, **B**). Nuclei were elongated. The cytoplasm was inconspicuous. Skin adnexa were spared (**B**). The Elastic stain highlights prominent elastic fibers throughout the lesion (**C**). SMA was focally expressed in case 5 (**D**)

**Fig. 2:**
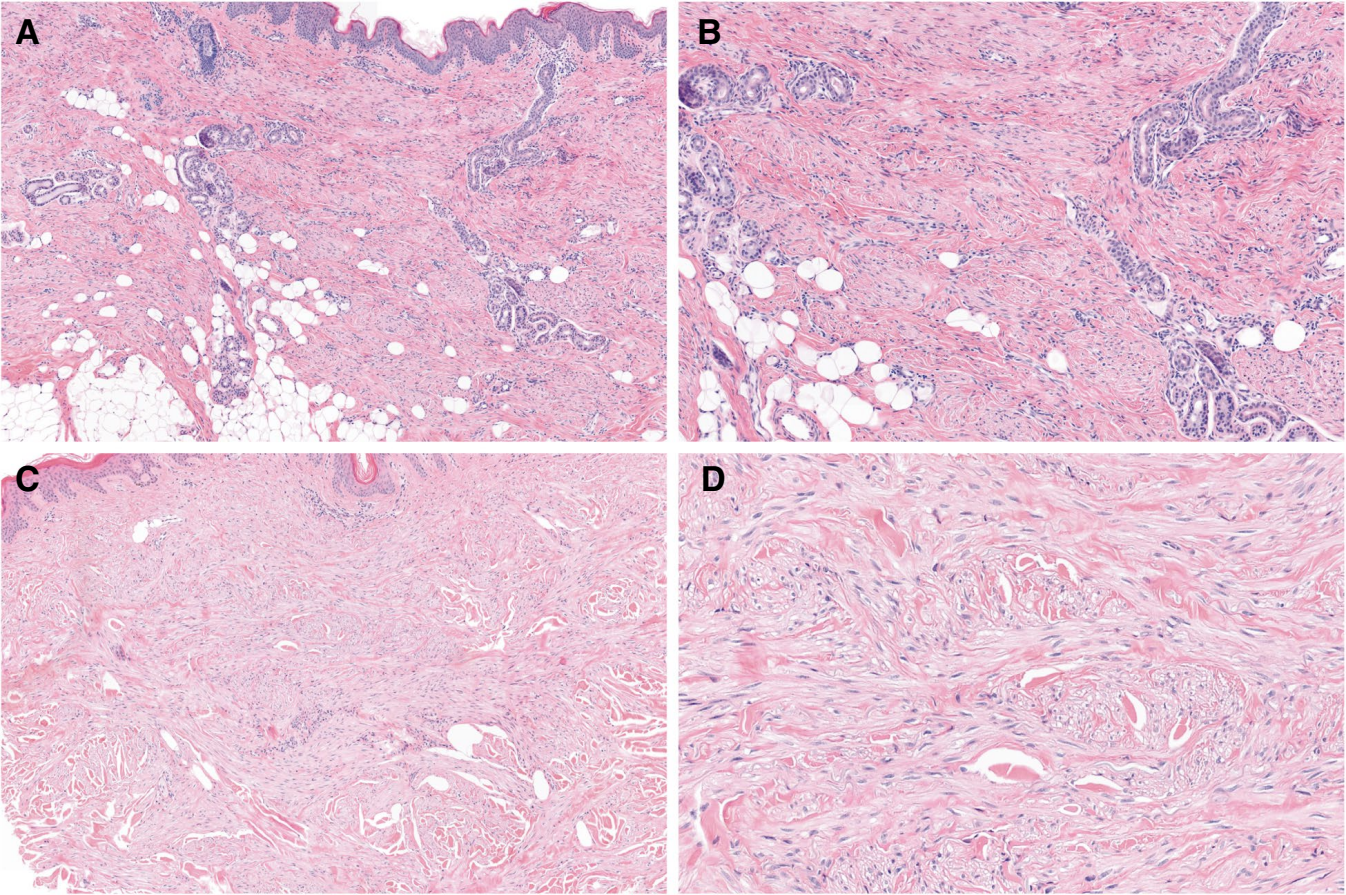
Case 4 (**A**, **B**) and case 7 (**C**, **D**) show similar histological characteristics, as demonstrated in Fig. 1

Histologically, tumors were confined to the dermis or infiltrated superficially into the subcutis (*n* = 4). They consisted of monomorphic, slender myofibroblastic cells arranged in bundles mainly parallel to the skin surface. The nuclei were elongated and possessed open chromatin. The cytoplasm was inconspicuous. Skin adnexa were spared (Fig. 1A, B Fig. 2). Excised neoplasms were totally removed.

Elastic stain, when performed, highlighted prominent and thick elastic fibers throughout the lesions (Fig. 1C).

By immunohistochemistry, SMA was partially positive in five instances (Fig. 1D) and negative in two. Desmin, performed in five neoplasms, was focally positive in one of them. Six tumors were stained for CD34, with four being focally positive and two negative. S100 was lacking in all seven cases.

**Fig. 3:**
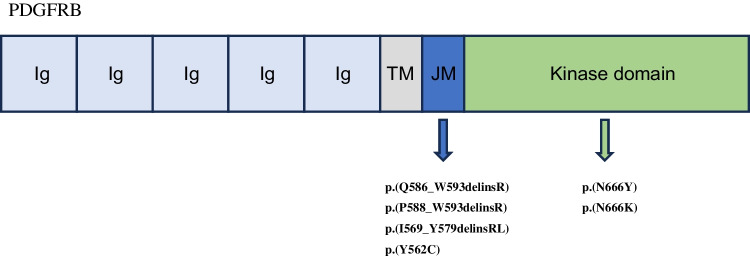
Structure of PDGFRB; Ig, immunoglobulin-like domains; TM; transmembrane domain; JM, juxtamembrane domain. As depicted, the identified mutations of our cases corresponded to the juxtamembrane and kinase domains

**Fig. 4:**
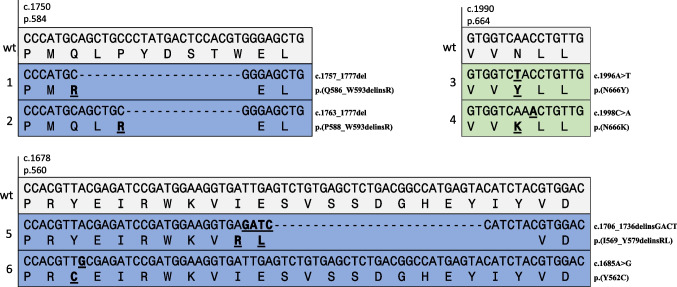
Details of the molecular aberrations. Wild-type (wt) cDNA and protein sequences of *PDGFRB* (NM_002609.4; GRCh37) compared to the mutated sequences detected in cases 1 to 6. Color coding of the associated domains is as in Fig. 2

DNA or RNA sequencing revealed a point mutation or in-frame delins mutation in *PDGFRB* in six out of seven cases. In four cases, the mutation occurred in exon 12, encoding for the juxtamembrane domain (p.(Q586_W593 delinsR), p.(P588_W593 delinsR), p.(I569_Y579 delinsRL), and p.(Y562 C)). Two cases harbored a hotspot mutation in exon 14, encoding for the kinase domain (p.(N666Y) and p.(N666 K)) (Figs. 2, 3 and 4). In one case, no *PDGFRB* mutation was found.

## Discussion

Dermatomyofibromas were first reported in 1991 by Huegel and in 1992 by Kamino et al. as a cutaneous plaque-like proliferation of fibroblasts and myofibroblasts in mainly young females with a predilection for the shoulder girdle [26, 27]. However, the age range is broad, including the pediatric population and even congenital neoplasms [28–30]. In a few cases, multifocality is reported [27, 29].

Histologically, neoplasms are ill-defined, consisting of uniform slender spindle-shaped (myo)fibroblasts arranged in well-defined elongated and intersecting fascicles, predominantly parallel to the skin surface. The cells possess tapering or elongated normochromatic nuclei and inconspicuous cytoplasm. Elastic fibers are preserved and may appear thicker than normal, which may serve as a diagnostic clue, especially when an elastic stain is available [26, 27, 30]. When infiltrating the subcutis, overlapping features with lipofibromatosis or lipofibromatosis-like neural lesions can be observed, including neuroid cytomorphology [10, 13, 27]. Sparing of skin adnexa is also very typical for dermatomyofibromas and other protein kinase (PK)–related tumors, including DFSP [27, 29]. Because of the histomorphological overlap with other myofibroblastic lesions, such as plaque-like myofibroblastic tumors, it has been debated whether, in fact, dermatomyofibromas are a separate entity [28].

Immunohistochemically, the (myo)fibroblastic phenotype is highlighted by variable expression of SMA and calponin, while desmin and h-caldesmon are commonly negative [26–28, 30]. A focal expression of CD34 may be observed [27, 30].

*PDGFRB*, located on 5q32, encodes the platelet-derived growth factor receptor beta, a cell surface tyrosine kinase for members of the platelet-derived growth factor family comprising PDGF A, B, C, and D acting as mitogens. Upon PDGF binding, receptor dimerization leads to autophosphorylation of tyrosine residues and activation of downstream signaling impacting proliferation, differentiation, survival, and migration of cells [11, 31, 32]. Constitutive activation of PDGFRB results, like in other RTKs, in elevated signal transduction of the PI3 K-AKT-mTOR pathway [4, 11, 13, 33]. Gain-of-function mutations and fusion genes induce change of receptor conformation and autonomous activation, which is reported in tumors with a myofibroblastic/pericytic phenotype. This includes the broad spectrum of pericytic tumors, hereditary and sporadic, inflammatory myofibroblastic tumors with malignant behavior (inflammatory myofibroblastic sarcoma), lipofibromatosis, and high-grade sarcomas with myogenic differentiation [5, 13–15, 17, 18, 31, 33–35].

In our cohort of dermatomyofibromas, six out of seven tumors harbored a *PDGFR* exon 12 or exon 14 mutation, which seems to be the driver of this (myo)fibroblastic neoplasm. The involved exons encode for the juxtamembrane and kinase domain, respectively, thereby influencing the kinase function. All the detected in-frame delins mutations in our study occurred in exon 12, likely abrogating the kinase inhibitory function of the juxtamembrane domain, thus leading to ligand-independent activation [32]. In contrast, the hotspot exon 14 mutations are suggested to result in constitutive activation by altering the tyrosine kinase domain itself into an active conformation [32]. Similar mutations are identified in the broad spectrum of pericytic tumors, including myofibromas and in high-grade sarcomas with myogenic differentiation [5, 18, 19, 35]. Myofibroma is also a morphological differential diagnosis. In contrast to dermatomyofibroma, it shows a (multi)nodular appearance and a biphasic pattern of more primitive cells accompanied by a hemangiopericytoma-like vasculature and mature myofibroblasts set in a typical bluish matrix [2, 27]. The overlapping morphological features with lipofibromatosis, lipofibromatosis-like neural tumors, and fibrous hamartoma of infancy (FHI) are not surprising, considering that all these lesions belong to the protein kinase–related group of tumors [9]. They have in common fascicles of monomorphic myofibroblastic cells, infiltrating subcutaneous fat, with FHI showing by definition additional nests of primitive cells. Other PK-related neoplasms in the differential diagnoses are the plaque-like stage of dermatofibrosarcoma protuberans (DFSP) and the *ALK*-rearranged CD34-positive spindle cell neoplasm/medallion-like dendrocyte hamartoma/plaque-like CD34-positive dermal fibroma. They are also characterized by horizontally orientated fascicles or diffusely arranged monomorphic (myo)fibroblastic cells but distinguished by a strong CD34 staining pattern with ALK co-expression due to *ALK* rearrangement in the latter tumor type [27, 36]. DFSP, instead, commonly harbors a *PDGF* fusion gene without receptor involvement [2]. The differential diagnoses scar and dermatofibroma do not spare skin adnexa and do not contain elastic fibers, in contrast to dermatomyofibroma. While scar tissue has a more haphazard appearance, dermatofibroma, consisting of fibrohistiocytic cells, shows mainly a radiating architecture with inclusion of broad dermal collagen at the edge [2, 27, 30].

Other differential diagnoses are neurofibroma (CD34, SOX10 and S100 +), perineurioma (EMA, CD34 and GLUT1 +), connective tissue nevus (more sclerotic, CD34 +, SMA -/+), blue nevus (melanocytic markers), fibroblastic subtype of plexiform fibrohistiocytic tumor/plexiform myofibroblastoma (SMA +, CD34 and desmin ±), pilar leiomyoma and smooth muscle hamartoma (smooth muscle markers), and plaque-like myofibroblastic tumor (short fascicles of myofibroblastic cells, wiry collagen bundles, SMA +, factor XIIIA +) [6, 27–30, 37].

Excision of dermatomyofibroma is not per se recommended as they can regress spontaneously, and incomplete excision does not lead to progression or recurrences. Stable disease was also seen in two of our patients, where the tumors were not excised [27, 29, 30].

In conclusion, dermatomyofibroma, a benign myofibroblastic tumor, harbors recurrent gain-of-function mutations in the *PDGFRB* gene*.* This leads to an active conformation of the receptor tyrosine kinase and oncogenesis. Dermatomyofibromas are, therefore, one example of the protein kinase–related tumors.
